# Comparative analysis of aroma profiles and determination of key constituents in organically and conventionally produced white wines

**DOI:** 10.1038/s41538-026-00811-w

**Published:** 2026-04-01

**Authors:** Tomáš Bajer, Pavel Šmejda, Jana Kubáleková, Petra Bajerová

**Affiliations:** Department of Analytical Chemistry, Faculty of Chemical Technology, University of Pardubice, Pardubice, Czechia

**Keywords:** Chemistry, Materials science

## Abstract

This study compares the aromatic profiles of Czech white wines produced under conventional and organic practices and identifies the volatile compounds most relevant for distinguishing these production systems. Seventy-eight wines (49 conventional, 29 organic) were analyzed by HS-SPME/GC-FID, with GC-MS used for compound identification, and data were evaluated using OPLS with the logarithm of likelihood ratio as the response variable. The calibration model (n = 122) explained 96.7% of the total variation and achieved 92.3% cross-validated predictability, with both sensitivity and specificity reaching 1.000 for conventional and organic wines; external validation (*n* = 34) confirmed perfect classification accuracy (RMSE = 0.17). Among the 20 most influential predictors, 16 volatile compounds were identified as significant markers. Organic wines exhibited substantially higher concentrations of malolactic fermentation indicators, with ethyl lactate and isoamyl lactate averaging 39-fold and 7-fold higher levels, respectively. Conversely, hexyl acetate was 9-fold more abundant in conventional wines, which aligned with the contrasting hexanol:hexyl acetate ratios (48.0 organic vs. 4.5 conventional). Organic wines also contained 29-fold higher caprylic acid, accompanied by a markedly different ethyl caprylate:caprylic acid ratio (280 vs. 20). These compositional patterns provide a robust chemical fingerprint enabling reliable differentiation of production systems.

## Introduction

Wine, a fermented beverage, has been an integral component of human civilization for millennia, transcending its role as merely an alcoholic drink to become a significant cultural artifact. The evolution of vinification technology, the enhancement of vintner education, and the escalating consumption of wine have collectively contributed to the refinement of wine quality and the diversification of wine varieties. This includes not only wines derived from conventional production methods but also those labeled as “organic”. Conventional production encapsulates the current standard vinification techniques employed by both boutique wineries and industrial wine manufacturers. Organic wines (natural wines), which has the first legal definition (in France) since 2020^1^, are generally characterized by consumer preference for sustainably cultivated wines, with minimal additives or interventions in both the vineyard and the winery.

The market for organic wines is witnessing a global upward trajectory. This surge is not solely attributed to the escalating demand from end consumers, but also their integration into the luxury gastronomy sector, owing to their distinct sensory attributes. The classification of wines as organic, biodynamic, natural, devoid of added sulfur dioxide, low environmental impact, vegan, or vegetarian is somewhat nebulous, with such wines being globally available^2^. These unconventional wine categories can be bifurcated into those adhering to legislative mandates or satisfying the criteria of a certification authority, and those produced entirely outside any regulatory framework. From a consumer perspective, the common denominator for these wines is the perceived advantage of a healthful, additive-free wine derived from environmentally conscious agricultural practices. A study conducted by Moscovici et al.^3^ demonstrated that respondents exhibited a willingness to pay a premium for organically labeled wine, irrespective of its country of origin.

The quality of wine is predominantly ascertained by its comprehensive organoleptic properties. Currently, no instrumental analytical methodology exists that can encapsulate the complexity of wine in the same manner as sensory analysis. Several studies have been published that focus on the characterization of organic wines or their comparative analysis with conventionally produced wines. Lante et al.^4^ conducted an investigation into Italian organic wines, examining certain chemical parameters (e.g., acidity, alcohol content, sugar levels, and phenolic compound content) and sensory characteristics. The research conducted by Sotnikova et al.^5^ focused on the color and sensory evaluation of six organic and six conventionally produced wines in the Czech Republic. The sensory evaluation results did not reveal significant differences between conventionally and organically produced wines, but varietal differences were observed. The study by Laureati et al.^6^ compared organic and conventional Sangiovese PDO (Protected Designation of Origin) wines from two consecutive vintages using sensory descriptors and instrumental parameters (such as total acidity, volatile acidity, pH, total polyphenol content, and free anthocyanin levels). Organic and conventional wines exhibited differences across nearly all instrumental parameters, while sensory differences were less distinct.

Additional studies have been published that amalgamate instrumental analysis and statistical evaluation of the obtained data to distinguish between conventional and organic wine types. Cozzolino et al.^7^ employed MIR spectroscopy in conjunction with multivariate analysis to classify commercial Australian wines originating from organic and non-organic production systems. Korenovska and Suhaj^8^ utilized elemental analysis combined with canonical discriminant analysis to compare Slovak wines from organic and conventional production. Significant differences in the representation of certain elements were observed. Moyano et al.^9^ integrated the determination of basic parameters (e.g., sugars, volatile and titratable acidity, pH, SO_2_), quantification of individual compounds, and odor activity values of odor-active compounds with principal component analysis to study differences in the aroma of young and biologically aged sherry wines from Pedro Ximenez grapes cultivated conventionally and organically in Spain.

Given the disparities in organic and conventional viticulture systems, coupled with the unique sensory attributes of organic wines, it can be postulated that the aromatic profiles of organic and conventional wines harbor distinguishing information. Nevertheless, even when employing suitable extraction methodologies in tandem with Gas Chromatography-Mass Spectrometry, the identification of a sufficient number of compounds for a comprehensive comparison of volatile profiles presents a challenge. Numerous compounds, potentially pivotal for such an analysis, remain unidentified and may consequently be omitted from the comparative study of different wine types^10,11^.

The aroma of wine is traditionally divided into three levels, which reflect the technological process of production. The primary (varietal) aroma comes directly from the grapes and is caused by, for example, terpenes, pyrazines, or carbonyl compounds^12–14^. The secondary (fermentation) aroma is created during alcoholic and malolactic fermentation (MLF) and includes the most numerous group of substances, such as esters, higher alcohols, fatty acids, and sulfur compounds^15,16^. Tertiary aromas (bouquet) develop during wine maturation (in barrels or bottles) as a result of oxidation, hydrolysis, or extraction of substances from wood, such as lactones, furanic compounds, and phenolic compounds^17–19^.

The Czech Republic is a traditional wine-growing region with a cool climate, where wine production is concentrated in two wine-growing areas (Moravia and Bohemia) covering approximately 18,000 hectares. The country’s variety composition is predominantly characterized by white varieties, which make up approximately 70% of the total vineyard area. Among them, the most widespread cultivars are Grüner Veltliner, Müller Thurgau, and Riesling. Red (blue) varieties account for 28.5% of the total area, with the main representatives being Blaufränkisch and St. Laurent. In addition, there is a noticeable and growing proportion of mold-resistant varieties, such as Hibernal, reflecting a shift towards sustainable viticultural practices in the region. This specific distribution of varieties, influenced by Central European climatic conditions, provides a solid basis for investigating the chemical markers and aromatic profiles associated with different farming systems. Understanding these basic characteristics is key to comparing the volatile composition of organic and conventional wines produced in this area^20^.

The objective of this study was to devise a methodology for the analysis of the aromatic profiles of white wines produced in Czech Republic, utilizing the complete aromatic profile as a distinguishing fingerprint between conventional and organic wines, and to subsequently identify the compounds indicative of a specific type of wine. In alignment with research pertaining to the quantification of aromatic substances in foodstuffs and beverages, HS-SPME and GC-FID were selected for the ascertainment of quantitative parameters, supplemented by a mass detector for compound identification. A similar approach, combining HS-SPME and GC-FID, has been successfully used, for example, to study the authenticity of fruit distillates^21^ or to distinguish the geographical origin of larch trees^22^. Chromatographic data were statistical evaluated via Orthogonal Projections to Latent Structures (OPLS). Significant compounds, which were instrumental in differentiating the volatile profiles of the two types of wines, were identified using mass spectrometry and verified by linear retention indices.

## Results and discussion

### Optimization of headspace solid-phase microextraction method

The central composite design (CDD) method was used to optimize the extraction time and temperature parameters of the HS-SPME technique. The measured data were evaluated using the response surface methodology (RSM). The extraction temperature was examined in the range of 30–90 °C, and the extraction time was examined from 10 to 120 min, with the number of peaks in each chromatogram as the response variable. These ranges were selected based on the tested matrix, and with the aim of extracting as many substances as possible. The results obtained are presented in Table 1.

**Table 1:** Design of the HS-SPME optimization experiment showing the extraction conditions and the corresponding results expressed as the total number of peaks in each chromatogram

Run	Temperature [°C]	Time [min]	Number of peaks	
			Observed	Predicted
1	30	10	74	78
2	30	90	107	108
3	30	50	101	99
4	60	10	94	91
5	60	90	120	123
6	60	50	114	113
7	60	50	116	113
8	60	50	111	113
9	60	50	115	113
10	90	10	73	75
11	90	90	105	109
12	90	50	98	97
13	30	120	111	108
14	60	120	120	123
15	90	120	115	110

Temperature range testing: 30–60 °C is the range in which highly volatile substances and esters are commonly analyzed to prevent their thermal degradation or hydrolysis. The upper limit of 90 °C is rarely used in wine aroma profile analysis (due to the risk of artifacts from the Maillard reaction or oxidation), but in this case it serves as a point at which it has been shown that increasing the temperature above 60 °C does not lead to better results.

A time range of up to 120 min is suitable for SPME in wine, especially when the goal (as in this case) is a complex aroma profile. Long times can lead to a displacement effect, where substances with a higher boiling point (but with a higher affinity for the fiber) gradually displace lighter substances. The fact that the optimum was 110 min suggests that the method is designed to maximize sensitivity even for these heavier substances, which is desirable for wine profiling.

**Fig. 1:**
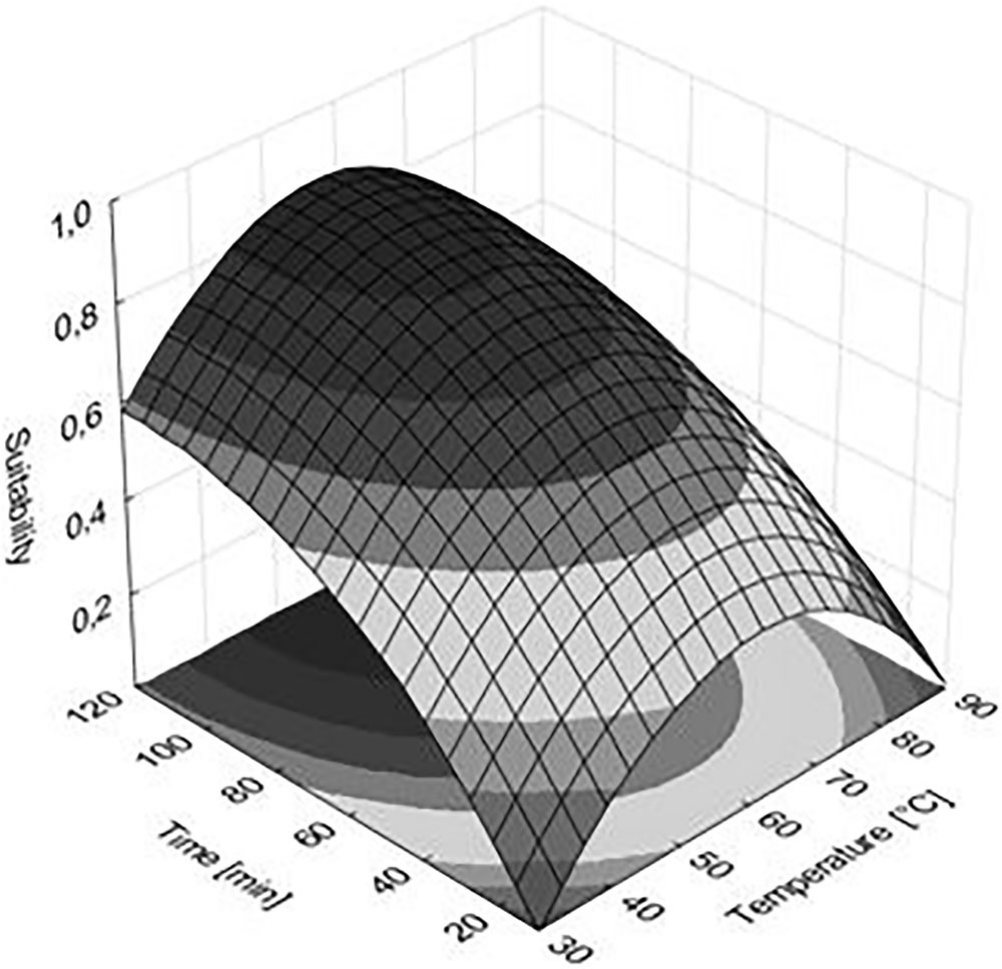
Response surface profile for predicting optimal extraction conditions; dark color means the area of optimal conditions.

The response surface was constructed (Fig. 1) based on the measured values of the number of peaks in the chromatograms, and the values of the extraction time and temperature. The dark region of the surface represents the optimal conditions. Consequently, an extraction temperature of 60 °C and an extraction duration of 110 min were determined as the optimal extraction conditions.

Statistical evaluation of the model was performed using analysis of variance (ANOVA, Table 2) with STATISTICA software, version 12 StatSoft CR (Prague, Czech Republic). The significance of each factor was tested using *p*-values and F-values. Factors a11 (quadratic temperature term), a2 (linear time term), and a22 (quadratic time term) have a statistically significant effect on extraction, as all their *p*-values are significantly lower than 0.05 (ranging from 0.0003 to 0.0040). This statistical validation confirms that both temperature and time had to be optimized to achieve maximum yield. Although the linear temperature term (a1) is not significant, the quadratic term (a11) is extremely significant (*F* = 190.47), which proves that a local maximum was found within the measured interval, confirming the correctness of the selected range of 30–90 °C. The *p*-value for the lack-of-fit test is 0.1401, which means that the model error is not statistically significant. The interaction between time and temperature was not significant (*p* = 0.3571). This means that the effect of temperature does not change with time and vice versa. The factors act independently, which simplifies process control. The RSM model is suitable for describing and predicting optimal extraction conditions within the given experimental range. The coefficient of determination (*R*²) is high (0.9591, adjusted 0.9364), indicating strong predictive power. The high F values of the above-mentioned factors (ranging from 64.51 to 407.25) show that the variability caused by these factors is much greater than the random error. The greatest influence on the result is the quadratic effect of temperature (a11) with *F* = 164.60 and the linear effect of time (a2) with *F* = 407.25. This suggests that the choice of temperature and time is the most important factor for a successful extraction process with the aim of profiling as many compounds as possible in the wine aroma.

**Table 2:** Regression coefficients of the model and analysis of variance of obtained results

Factor	Regression coefficient	Standard error	DF	SS	*F*-value	*p*-value
a_0_	27.6164	5.0728				
a_1_	1.9262	0.1618	1	0.717	0.15	0.7212
a_11_	−0.0165	0.0013	1	768.129	164.60	0.0010
a_2_	0.7173	0.0699	1	1900.489	407.25	0.0003
a_22_	−0.0035	0.0004	1	301.066	64.51	0.0040
a_12_	0.0007	0.0006	1	5.500	1.18	0.3571
Lack-of-fit			6	112.770	4.03	0.1401
Pure error			3	14		

*DF* degrees of freedom; *SS* sum of squares.

Based on the ANOVA analysis, it can be concluded with high reliability that the model is statistically valid and that the tested parameters (temperature and time) are key factors for the final extraction yield. The coefficient of determination and the lack-of-fit test confirm that the model is a good predictor in this HS-SPME experiment. The mathematical Eq. (E1) describing the model is as follows:

E1 $$Z=27.6164+1.9262x-0.0165{x}^{2}+0.7173y-0.0035{y}^{2}+0.0007$$

where *Z* is the number of detected peaks, *x* is the extraction temperature, and *y* is the extraction time.

### Classification analysis

Using the procedures described in the “Data analysis” section and the “Calibration and validation of OPLS model” subsection, an OPLS model was derived that consists of five components: one predictive and four orthogonal. The predictive component encapsulates information in X that is predictive to Y. The orthogonal components express information exclusive to X, i.e., they represent systematic information that is unique in X (orthogonal in Y).

The model accounted for 96.7% of the variation in the training set, as explained by the predictive and orthogonal components. This is a measure of fit, i.e., the degree to which the model aligns with the data. The predicted variation of the training set, as per cross-validation, is 92.3%, indicating the model’s robust predictive capability for new data.

The derived OPLS model demonstrated excellent discrimination, with a sensitivity of 1.000 (0.952, 1.000) and a specificity of 1.000 (0.916, 1.000) for predicting the conventional type of wine in the tested sample set (estimates within 95% confidence limits). This implies that all positive subjects (samples of conventional wines) were correctly identified as positive, with no negative subjects falsely identified as positive. The model’s prediction of organic wines mirrored this, with a sensitivity of 1.000 (0.916, 1.000) and a specificity of 1.000 (0.952, 1.000).

**Fig. 2:**
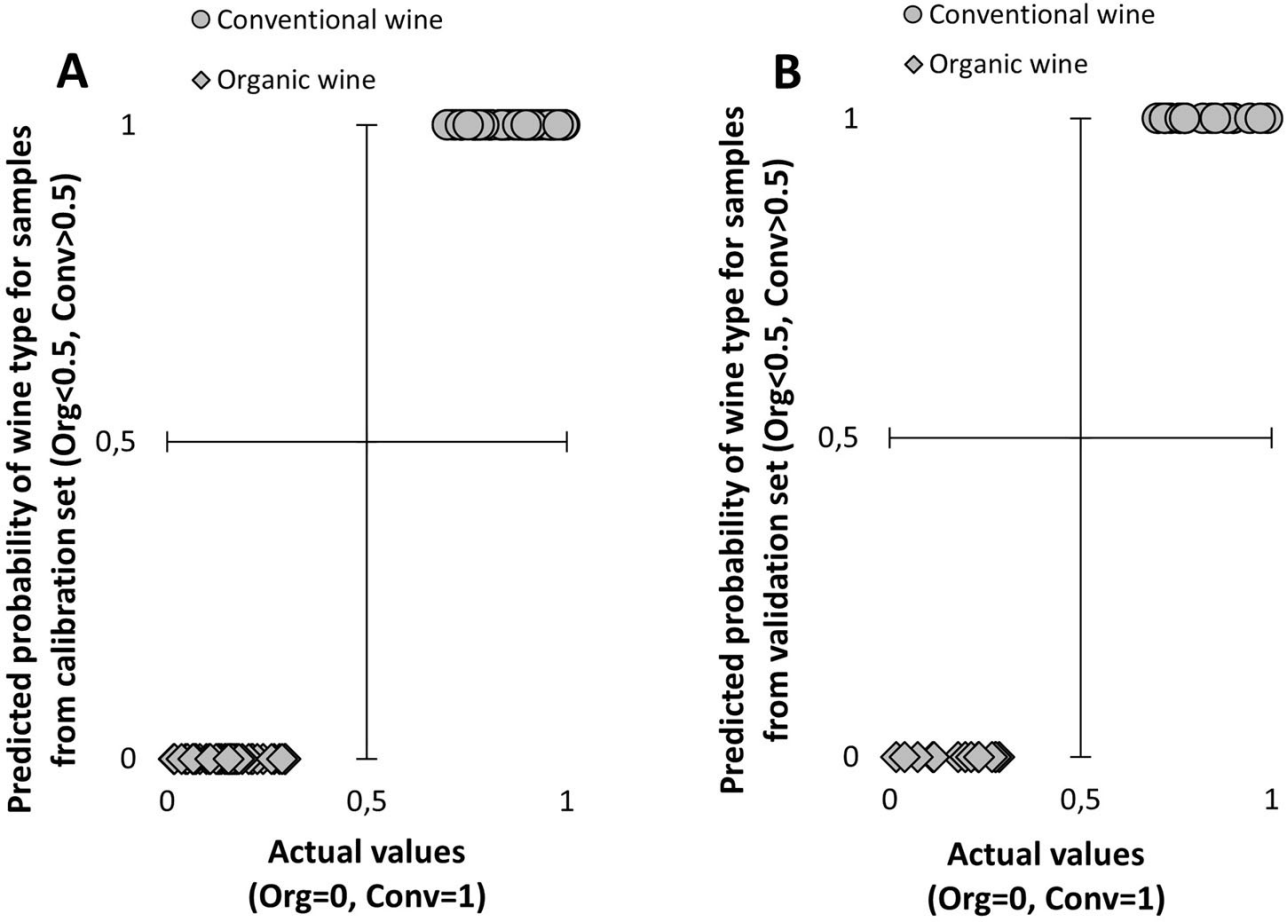
Comparison of predicted probability of kind of wine and actual values. Samples from **A** work (calibration) dataset, **B** validation dataset. RMSE (**A**) = 0.17, RMSE (**B**) = 0.17. The variance of the actual values (0 for organic wines, 1 for conventional wines) is given by jittering.

Figure 2A illustrates a comparison of the calculated probability value that a subject from the calibration dataset is a sample of conventional (or organic) wine against the actual value. The root mean square error (RMSE), a measure of the differences between observed values and values calculated from the model’s prediction, is 0.17.

The model’s predictability was further validated using a validation dataset (data of samples not included in the calibration dataset, see section “Data analysis in materials and methods”). Results are depicted in Fig. 2B, where the calculated probability value that a subject of the validation dataset is a sample of conventional (or organic) wine is compared to the actual value. All 34 observations from the validation set were accurately classified, with an RMSE value of 0.17.

The presented data demonstrate that the chromatographic information (chromatographic peaks distinguished by retention indices and peak areas) procured via HS-SPME/GC-FID can serve as a distinctive identifier for both natural and conventional white wines, thereby facilitating the verification of their origin. Certain volatiles, particularly their combinations, can significantly contribute to authenticity verification. The data also underscore the importance of selecting appropriate parameters for the extraction method.

### Identification of relevant predictors

**Fig. 3:**
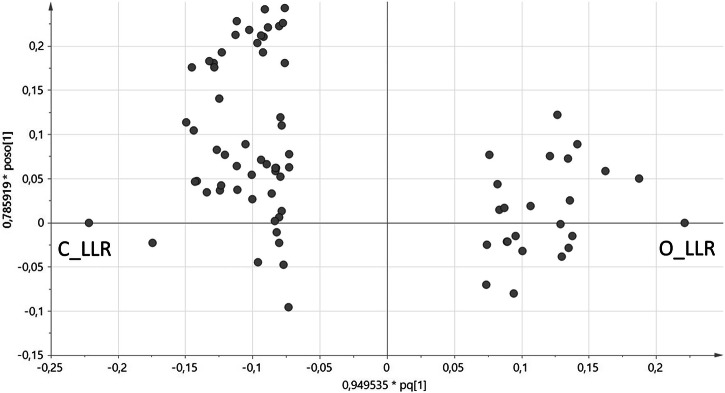
Loadings scatter plot shows relationship between *X*- and *Y*-variables for predictive component (*X*-axis) and the 1st orthogonal component (*Y*-axis). C_LLR… logarithm of the likelihood ratio (logarithms of the ratios of the probability that the subject is a conventional wine to the probability that the subject is a control=organic wine) O_LLR… logarithm of the likelihood ratio (logarithms of the ratios of the probability that the subject is an organic wine to the probability that the subject is a control=conventional wine).

Depending on Fig. 3, it follows that a greater number of predictors are correlated with the dependent variable on the left side (C_LLR), indicating that a larger proportion of compounds in the calibration model serve as predictors for conventional wine (53 out of 76) compared to organic wines (23 out of 76).

Table 2 delineates the relationships between the predicted variable (wine type) and the pertinent predictors (volatile compounds, characterized by retention indices) using OPLS and MR. Twenty predictors exhibiting the highest correlation with the Y variable were selected (10 associated with conventional wines and 10 associated with organic wines). These are the relevant predictors whose correlation with the dependent variable is independent of the other predictors. For 16 out of the 20 chosen predictors, compound names were proposed based on comparisons of mass spectra and retention indices (RI) with published data (databases: 1) Flavor and Fragrance Natural and Synthetic Compounds, FFNSC2; 2) NIST webbook (William E. Wallace, “Retention Indices” in NIST Chemistry WebBook, NIST Standard Reference Database Number 69)), and in some instances, standard solutions. Detailed information (mass spectra of predictors, calculated retention indices, and retention indices from the databases) is provided in Table 3 and Supplementary Figs. 1 and 2.

**Table 3:** Relationships between 20 selected predictors (peaks with calculated retention indices; selected according to the impact on the predictive component) and the kind of wine for the predictive component evaluated by OPLS and by MR (relationships between LLR and predictors)

							OPLS predictive component				Multiple regression					
											C_LLR			O_LLR		
Selected relevant predictors (matrix **X**)	Code of predictor	Proposed compound	CAS	Method of identification^a^	Calculated RI	RI from database	Component loadings	*t*-statistic	R^*f*^		Regression coefficients	*t*-statistic		Regression coefficients	*t*-statistic	
	RI0813	Ethyl lactate	97-64-3	MS, RI	813	814^b^	0.145	7.4	0.632	**	−0.110	−4.30	**	0.110	4.30	**
	RI0878	Isoamyl acetate	123-92-2	MS, RI	878	876^c^	0.078	2.6	0.340	*	−0.054	−2.48	*	0.054	2.48	*
	RI0879	2-methylbutyl acetate	624-41-9	MS, RI	879	880^d^	0.142	5.5	0.618	**	−0.092	−4.79	**	0.092	4.79	**
	RI0969	Linaloyl oxide	7392-19-0	MS, RI	969	968^b^	0.128	6.3	0.556	**	−0.120	−3.95	**	0.120	3.95	**
	RI0974	Heptanol	111-70-6	MS, RI, S	974	970^b^	0.092	4.4	0.400	**	−0.136	−4.97	**	0.136	4.97	**
	RI1013	Hexyl acetate	142-92-7	MS, RI	1013	1012^b^	−0.183	−13.9	−0.800	**	0.059	4.52	**	−0.059	−4.52	**
	RI1018	---	---	---	1018	---	−0.136	−6.1	−0.592	**	0.053	4.75	**	−0.053	−4.75	**
	RI1032	Limonene	138-86-3	MS, RI, S	1032	1030^b^	−0.139	−8.0	−0.606	**	0.108	4.70	**	−0.108	−4.70	**
	RI1037	(Z)-β-Ocimene	3338-55-4	MS, RI, S	1037	1035^b^	−0.151	−23.2	−0.659	**	0.059	3.82	**	−0.059	−3.82	**
	RI1061	γ-Terpinene	99-85-4	MS, RI, S	1061	1058^b^	−0.135	−8.1	−0.589	**	0.085	2.08	*	−0.085	−2.08	*
	RI1069	Isoamyl lactate	19329-89-6	MS, RI	1069	1067^e^	0.171	11.2	0.746	**	−0.075	−2.84	*	0.075	2.84	*
	RI1071	(Z)-Linalool furanoxide	5989-33-3	MS, RI	1071	1069^b^	0.197	12.6	0.861	**	−0.137	−4.51	**	0.137	4.51	**
	RI1183	Caprylic acid	124-07-2	MS, RI	1183	1180^d^	−0.099	−9.5	−0.430	**	−0.097	−4.40	**	0.097	4.40	**
	RI1257	2-phenylethyl acetate	103-45-7	MS, RI	1257	1257^b^	−0.081	−3.1	-0.353	**	0.103	4.16	**	-0.103	−4.16	**
	RI1278	p-Ethylguaiacol	2785-89-9	MS, RI	1278	1275^b^	0.133	6.2	0.581	**	−0.106	−3.46	**	0.106	3.46	**
	RI1299	---	---	---	1299	---	−0.106	−6.4	−0.460	**	0.097	3.40	**	−0.097	−3.40	**
	RI1316	p-Vinylguaiacol	7786-61-0	MS, RI	1316	1312^d^	−0.082	−5.6	−0.359	**	0.098	2.82	*	−0.098	−2.82	*
	RI1454	---	---	---	1454	---	−0.086	−3.9	-0.376	**	0.073	3.44	**	−0.073	−3.44	**
	RI1483	---	---	---	1483	---	−0.088	−5.1	−0.384	**	−0.074	-4.01	**	0.074	4.01	**
	RI1615	Myristaldehyde	124-25-4	MS, RI	1615	1614^b^	−0.084	−7.8	−0.365	**	0.088	3.74	**	-0.088	−3.74	**
Predicted variable (matrix **Y**)	Conventional wine, C_LLR^*g*^						−0.233	−11,76	−0.983	**						
	Organic wine, O_LLR						0.233	11,76	0.983	**						

*p < 0.05

**p < 0.01

^a^MS…mass spectrum, RI…retention index, S…standard.

^b^RI from database Flavor and Fragrance Natural and Synthetic Compounds (FFNSC 2).

^c^Pino, J. A., Marbot, R., Rosado, A. & Vázquez, C. Volatile constituents of Malay rose apple [Syzygium malaccense (L.) Merr. & Perry]. Flavor Fragrance J. **19**, 32–35 (2004). 10.1002/ffj.1269.

^d^Pino, J. A., Mesa, J., Muñoz, Y., Martí, M. P. & Marbot, R. Volatile Components from Mango (Mangifera indica L.) Cultivars. J. Agric. Food Chem. **53**, 2213–2223 (2005). 10.1021/jf0402633.

^e^Thompson Witrick, K., Duncan, S. E., Hurley, K. E. & O’Keefe, S. F. Acid and Volatiles of Commercially-Available Lambic Beers. Beverages **3**, 51 (2017). 10.3390/beverages3040051.

^f^R…component loadings expressed as a correlation coefficients with predictive component.

^g^C_LLR… logarithm of the likelihood ratio (logarithms of the ratios of the probability that the subject is a conventional wine to the probability that the subject is not a conventional wine).

O_LLR… logarithm of the likelihood ratio (logarithms of the ratios of the probability that the subject is an organic wine to the probability that the subject is not a organic wine).

The average peak areas of all 20 predictors and other compounds discussed in the text below, expressed as relative percentages of the total peak area for comparison purposes, are given in the Supplementary Table 1. The calculation was performed using the area normalization method, whereby the areas in the chromatogram up to 3.00 min, when the ethanol peak elutes, which has a much larger area than the other analytes, were not included in the total peak area. Ethanol was not a target substance in this work and is not significant for the analysis of the aroma profile.

Of the selected relevant predictors, seven were identified as esters, compounds that are abundantly present in wine aroma profiles. Two of these were lactates, specifically ethyl lactate and isoamyl lactate. Lactates are produced in wines that have undergone MLF^23–26^, a process occurring in the presence of lactic acid bacteria. This process involves the conversion of sharp malic acid into smoother lactic acid and carbon dioxide. For the breakdown of organic acids, lactic bacteria must be robust and viable, a state challenging to attain in the presence of high SO_2_ concentrations. Ethyl lactate was identified in all organic wine samples as well as in 19 conventional wines. Isoamyl lactate was detected in all organic wines as well as in 17 conventional wine samples. The chromatograms of organic wines exhibited average peak areas of ethyl lactate that were three and a half times higher than those in conventionally produced wines. For isoamyl lactate, an even larger discrepancy was observed between organic and conventional wines, with a five-fold higher average peak area for organic wines. Hexyl acetate, another significant predictor, has precursors in yeast fermentation that are C6 alcohols and aldehydes, specifically hexanol, hexanal, (E)-2-hexenal, and (E)-2-hexen-1-ol^27,28^. Controlled yeast fermentation seems to yield significantly higher amounts of hexyl acetate, as the average peak area of hexyl acetate was nine times higher in chromatograms of conventionally produced wines than in organic wines. This is also suggested by the fact that the ratio of the average peak areas of hexanol to hexyl acetate is ten times higher in organic wines than in conventional wines (ratio of average peak areas (hexanol:hexyl acetate) 48 and 4.5, respectively), while the average peak areas of hexanol are comparable in the measured volatile profiles of conventional and organic wines. The significantly lower amount of hexyl acetate in organic wines may also be due to the effect of MLF, which was also observed for Tannat wines in the work of Gambaro^23^.

A notable difference was also found for caprylic acid, with the average peak area being almost 30 times higher for conventional wines. This finding may be attributed to the fact that C8 acid is easily biodegradable and therefore, may be less abundant in the volatile profile of organic wines due to the higher microbial diversity. And although ethyl caprylate is 2 times more abundant in the volatile profile of conventional wines, the average peak area ratio of ethyl caprylate to caprylic acid shows a significant difference between conventional and organic wines (ratio of average peak areas (ethyl caprylate : caprylic acid) 20 and 280, respectively).

Overall, the results of this investigation demonstrate that the volatile compound spectra of organically and conventionally produced white wines contain sufficient discriminative information to reliably distinguish between the two production systems. The HS‑SPME/GC‑FID chromatographic data, evaluated using OPLS modeling, revealed clear and reproducible chemical differences between wine types that were consistent across both calibration and external validation datasets.

A total of 78 white wine samples underwent analysis via Headspace Solid‑Phase Microextraction and Gas Chromatography with a Flame Ionization Detector. The acquired data facilitated the construction of a statistical OPLS model, which successfully differentiated the wines into conventional and organic categories with perfect sensitivity and specificity within 95% confidence limits. The model’s robustness was confirmed through external validation, where all assayed samples were correctly classified, demonstrating that the volatile profiles contain embedded information reflective of the production system used.

Employing multiple regression without dimensionality reduction, in conjunction with further chromatographic analyses via mass spectrometry, enabled the identification of compounds exhibiting strong correlation with wine type. These included markers associated with MLF, esters derived from yeast metabolism, and fatty acid–related compounds. Their combined patterns highlight fundamental differences in winemaking practices and microbial activity, forming a reliable chemical fingerprint that contributes to the authentication and characterization of organically and conventionally produced Czech white wines.

## Methods

### Samples

A comprehensive set of 78 samples, encompassing both conventionally produced and organic white wines from the Czech Republic, were procured via a standard retail network and directly from vintners. The sample collection targeted bottled wines classified under the quality tiers of “Quality Varietal Wines” and “Quality Predicate Wines”. Organic wines were predominantly categorized by vintners as “Land Wines”, despite their price points aligning with ‘Quality Predicate Wines’ or higher.

For the scope of this study, the organic wine cohort incorporated wines that the vintner personally designates as organic or authentic, or those that employ unconventional viticultural practices (such as the elimination of spraying, extended maceration, minimal additions of sulfur dioxide, and coarse filtration only). Many of the organic wines in this study were sourced from vintners who have been engaged in this mode of production for several years, thereby representing a representative sample of wines in the Czech Republic that can be classified as organic. More granular classifications, such as the material of the fermentation or wine ageing container, were not considered in this study.

Within the set of 78 wines originating from two wine regions (Table 4), 49 are classified as conventionally produced and 29 as organic wines. Owing to the limited availability of organic wines in the market, the study incorporated different wine varieties with the intention of encompassing as much variability as possible.

**Table 4:** List of samples and representation of varieties; C…conventional production, O…organic production, Ce…Čechy Wine Region (Bohemia Wine Region), Mo…Morava Wine Region (Moravia Wine Region)

Sample Nr.	Kind of wine, Wine Region	Grape variety	Sample Nr.	Kind of wine, Wine Region	Grape variety
1	C, Mo	Müller-Thurgau	40	O, Mo	Gewürztraminer
2	C, Mo	Müller-Thurgau	41	O, Mo	Chardonnay
3	C, Mo	Müller-Thurgau	42	O, Mo	Sauvignon Blanc
4	C, Mo	Chardonnay	43	O, Mo	Cuvée
5	C, Mo	Grüner Veltliner	44	O, Mo	Riesling
6	C, Mo	Riesling	45	O, Mo	Cuvée
7	C, Mo	Riesling	46	C, Mo	Chardonnay
8	C, Mo	Grüner Veltliner	47	C, Ce	Riesling
9	C, Mo	Grüner Veltliner	48	C, Mo	Grüner Veltliner
10	C, Ce	Pinot Gris	49	O, Mo	Pinot Gris
11	C, Ce	Müller-Thurgau	50	O, Mo	Pinot Gris
12	C, Ce	Riesling	51	C, Mo	Pinot Gris
13	C, Ce	Riesling	52	C, Mo	Frühroter Veltliner
14	C, Ce	Moravian Muscat	53	C, Mo	Chardonnay
15	C, Ce	Riesling	54	C, Mo	Müller-Thurgau
16	C, Ce	Müller-Thurgau	55	C, Mo	Riesling
17	C, Ce	Riesling	56	C, Mo	Riesling
18	C, Ce	Riesling	57	O, Mo	Chardonnay
19	C, Ce	Müller-Thurgau	58	O, Mo	Chardonnay
20	N, Ce	Riesling	59	O, Mo	Gewürztraminer
21	C, Mo	Riesling	60	O, Mo	Cuvée
22	C, Mo	Riesling	61	C, Mo	Riesling
23	C, Mo	Chardonnay	62	C, Mo	Sylvaner
24	C, Ce	Müller-Thurgau	63	C, Mo	Grüner Veltliner
25	C, Ce	Bacchus	64	O, Mo	Sylvaner
26	C, Ce	Moravian Muscat	65	O, Mo	Müller-Thurgau
27	C, Ce	Sylvaner	66	C, Mo	Grüner Veltliner
28	C, Ce	Pinot Blanc	67	C, Mo	Riesling
29	C, Mo	Riesling	68	O, Mo	Neuburg
30	C, Mo	Grüner Veltliner	69	O, Mo	Grüner Veltliner
31	C, Mo	Chardonnay	70	O, Mo	Chardonnay
32	C, Mo	Neuburg	71	O, Mo	Pinot Gris
33	C, Mo	Müller-Thurgau	72	O, Mo	Grüner Veltliner
34	C, Mo	Riesling	73	O, Mo	Cuvée
35	C, Mo	Riesling	74	O, Mo	Sauvignon Blanc
36	N, Mo	Cuvée	75	O, Mo	Welschriesling
37	N, Mo	Cuvée	76	O, Mo	Cuvée
38	N, Mo	Cuvée	77	O, Mo	Sauvignon Blanc
39	N, Mo	Chardonnay	78	C, Mo	Chardonnay

### Chemicals and materials

The SPME fibers (supplied by Supelco, Bellefonte, PA, USA) used were StableFlex with a tickness of 50/30 μm with composition of DVB/CAR/PDMS (divinylbenzene/carboxen/polydimethylsiloxane). Analytical grade sodium chloride was procured from Lach-Ner s.r.o. (Neratovice, Czech Republic). The C8-C33 *n*-alkane standard solution was obtained from CPAchem (Stara Zagora, Bulgaria). The following individual volatile compounds were used: Heptanol, Limonene, (Z)-β-Ocimene, γ-Terpinene. These compounds were sourced from Merck (Darmstadt, Germany).

### Headspace solid-phase microextraction method

The experimental parameters for the headspace solid-phase microextraction (HS-SPME) method were optimized. The procedure was as follows: A 20 mL vial was filled with a mixture of 2.5 mL of wine and 7.5 mL of sodium chloride aqueous solution (28.5% (w/v)). Dilution with saline solution was chosen to mitigate the matrix effect of ethanol. Higher concentrations of ethanol (typical for wine matrices) act as a cosolvent, reducing the distribution coefficient of volatile compounds into the headspace and competing for active sites on the SPME fiber. Dilution, combined with the salting-out effect, significantly increases the ionic strength and promotes the release of hydrophobic analytes into the headspace, thereby improving the sensitivity of the method. The contents of the vial were thoroughly mixed. The vial was then sealed with a Teflon septum cap. To ensure steady-state extraction conditions, the samples were pre-incubated at a temperature of 60 °C for a duration of 20 min. The extraction of volatile compounds was performed using a 50/30 μm DVB/CAR/PDMS fiber, maintained at a temperature of 60 °C for a period of 110 min. Subsequently, the extracted volatile compounds were automatically desorbed in the GC injection port, which was set at a temperature of 200 °C, and fiber was subsequently cleaned using a cleaner system after desorption at 250 °C for 10 min.

### Chromatographic analysis

A Shimadzu (Kyoto, Japan) GC-2010 gas chromatograph equipped with a flame-ionization detector (FID) and GC-2010 mass spectrometer (QP 2010 Plus) was employed for the separation of volatile compounds. An autosampler, specifically the PAL-Combi from CTC Analytics (Zwingen, Switzerland), was utilized for the automated headspace solid-phase microextraction procedure. The separation was carried out on an SLB-5ms capillary column (30 × 0.25 mm; 0.25 μm of film thickness) from Supelco (Bellefonte, PA, USA). Helium 5.0 (supplied by Linde Gas a.s., Prague, Czech Republic) served as the carrier gas, maintaining a constant linear velocity of 30 cm s^−1^. The injector temperature was maintained at 200 °C, and the desorption time was 15 s. Injections were performed in split mode with a split ratio of 1:20. The column temperature program followed these steps: an initial temperature hold at 40 °C for 3 min, followed by a gradual increase up to 250 °C at a rate of 5 °C/min, held for 25 min. To ensure data reliability, each sample underwent duplicate analysis.

The operational parameters for the flame-ionization detector were configured as follows: The detector temperature was set to 270 °C. Hydrogen gas flowed through the detector at a rate of 40 mL min^−1^, while the air flow rate was maintained at 400 mL min^−1^. Additionally, nitrogen served as the make-up gas, flowing at a rate of 30 mL min^−1^.

The operational parameters of the mass spectrometer were as follows: both the interface and the ion source were maintained at a temperature of 200 °C. The *m/z* was scanned from 35 to 500, utilizing the electron ionization mode at an energy level of 70 eV.

A standard mixture of *n*-alkanes (C8-C33) was subjected to analysis under identical chromatographic conditions as the samples. Retention times of the *n*-alkanes were utilized for the calculation of the retention indexes (RI).

### Data analysis

The conditions for HS-SPME were meticulously optimized utilizing version 14.0 of the Statistica software, developed by TIBCO Software Inc. (Palo Alto, CA, USA). The task of classification analysis was executed with the assistance of the statistical software SIMCA, version 13.0, product of Sartorius Stedim Data Analytics AB (Umeå, Sweden).

Before conducting the statistical analysis, individual GC-FID chromatograms were scrutinized and retention indices for each peak were computed. The gathered data were transformed into a matrix X (r x c), where r represents rows (individual sample measurements) and c represents columns (calculated retention indices of peaks in chromatograms with corresponding peak areas). The calculation of retention indices was performed using the van Den Dool and Kratz formula^29^. The matrix was randomly bifurcated into two subsets: a calibration dataset and a validation dataset. The calibration model utilized 122 measurements (61 samples), while the validation model employed 34 measurements (17 samples).

The dimensionality-reducing multivariate regression method, Orthogonal Projections into Latent Structures (OPLS), was utilized to interpret the correlations between chromatographic data and the classification of wines as either conventional or organic. This method is equipped to handle multicollinearity (extensive intercorrelation) within the predictor matrix, a task unachievable by standard multiple regression, thereby enhancing the model’s predictive power.

The OPLS model partitions the systematic variation in X into two components: one that predicts Y (the predictive variation) and one that is orthogonal to Y. The predictive components model the X/Y predictive variation, while the orthogonal components model the variation in X that is orthogonal to Y. Variable Importance in Projection (VIP) statistics were employed to identify predictors pertinent to the analysis. This method is instrumental in pinpointing which attributes have significantly contributed to class differentiation.

In our OPLS models, the dependent variable Y was chosen as the logarithm of the likelihood ratio (LLR), which is the ratio of the probability that the subject belongs to a specific wine type to the probability that the subject belongs to another type. The predictors were individual peaks, labeled by the value of retention indices with peak area. LLR values were computed according to Eq. (E2). The actual LLR values theoretically range from –∞ to + ∞, but for practical evaluation, positive or negative numerical values were used. All objects of the investigated wine type have a positive LLR value, and all objects that do not belong to the investigated wine type have a negative LLR value. In this case, the class threshold is defined as LLR = 0.

E2 $$\mathrm{LLR}=\mathrm{ln}\left(\frac{\mathrm{A}}{\mathrm{B}}\right)$$

A… numerically expressed probability that the object belongs to the given category

B… numerically expressed probability that the object does not belong to the given category

### Calibration and validation of OPLS model

Initially, the data in calibration dataset were manipulated to achieve a symmetric distribution. To segregate uncorrelated information, two statistical methods were employed:

(1) The multivariate t-test (Hotelling’s T2 statistic) was used to confirm homogeneity among predictors. T2 values denote the distance of each observation from the origin in the model plane (score space).

(2) The Variable Importance for the Projection (VIP) was utilized to test the relevance of predictors. VIP values signify the significance of variables in explaining X and correlating to Y. X-variables with values greater than 1 are deemed relevant, while those with values less than 0.5 are considered “unimportant”. Variables with VIP values between 0.5 and 1 have varying importance depending on the dataset size (as per the SIMCA software manual). The VIP value > 1 is frequently used as a cut-off point for variable selection. However, due to the diversity and variability of data structures, the cut-off point may differ across different data structures. Determining the appropriate cut-off point is complex, as a value too high can eliminate key variables, and a value too low can include irrelevant variables in the analysis^30^. In our study, we used 0.5 as the cut-off point, and variables with VIP values outside the 95% confidence limit were disregarded.

Post the segregation of uncorrelated information, the ensuing parameters were computed: (a) component loadings for individual variables to assess the relationships between predictors and wine type, (b) regression coefficients for the multiple regression (MR) model to evaluate the relationships between predicted variables (LLR) and predictors (this is a specific predictor effect that is independent of the remaining predictors), (c) predicted LLR values and predicted probability of wine type for all observations, (d) sensitivity and specificity of the discrimination, (e) values of predicted probability.

## Supplementary information

Supplementary Information

## Data Availability

The datasets generated and/or analyzed during the current study are not publicly available due to the inclusion of commercially sensitive information provided by individual wine producers, but are available from the corresponding author on reasonable request.
