# Supplementary material for: Comparative analysis of aroma profiles and determination of key constituents in organically and conventionally produced white wines
Source: NPJ Sci Food. 2026 Apr 1;10:166. doi: 10.1038/s41538-026-00811-w (PMC13212730; doi:10.1038/s41538-026-00811-w)
Supplement: Supplementary file 1 — Supplementary Information [file 41538_2026_811_MOESM1_ESM.pdf]

Supplementary Figures 1 Measured mass spectra of all predictors listed in Table 3 of manuscript.

Ethyl lactate, similarity MS 99%, RI 813 (calculated), RI 814 (verified)

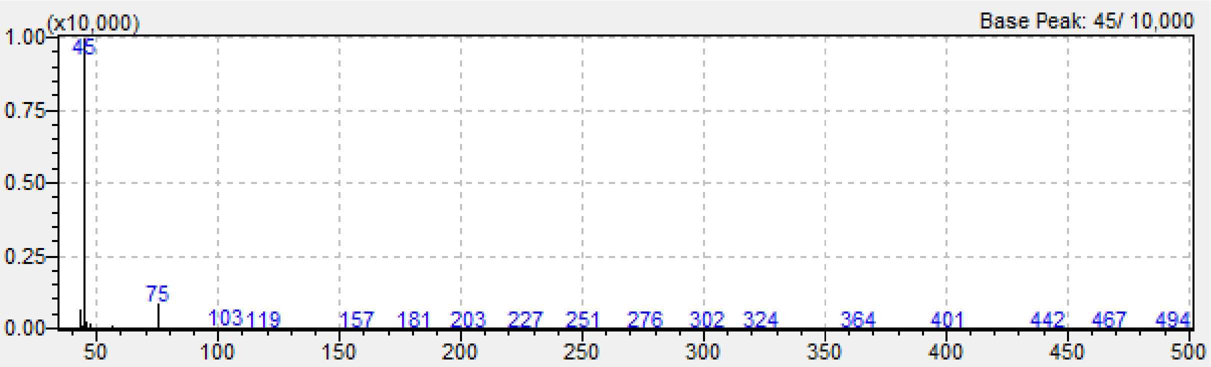

Isoamyl acetate, similarity MS 98%, RI 877 (calculated), RI 876 (verified)

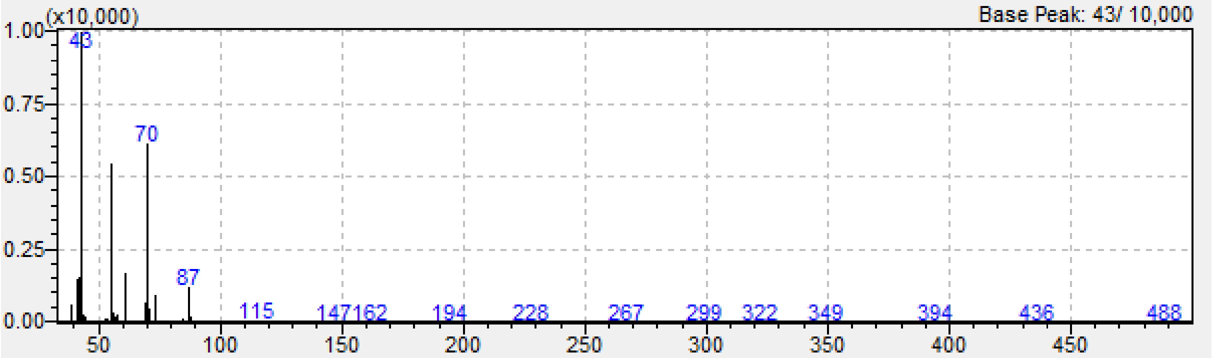

2-methylbutyl acetate, similarity MS 91%, RI 879 (calculated), RI 880 (verified)

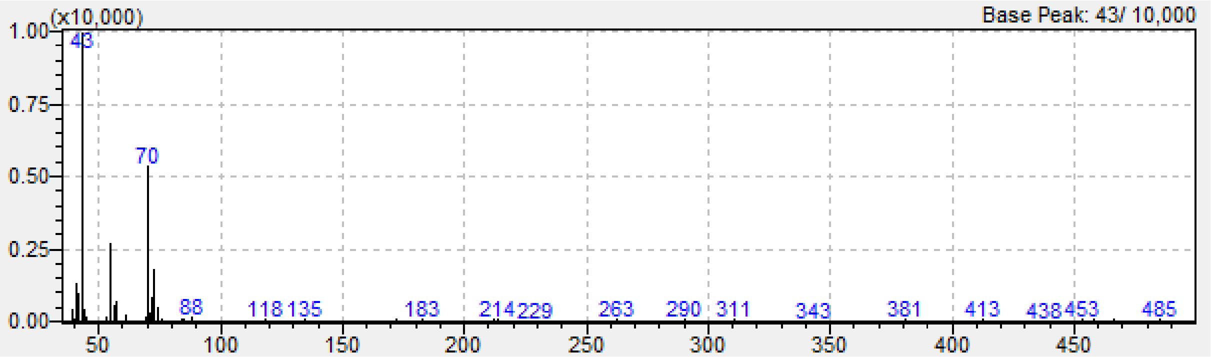

Linaloyl oxide, similarity MS 94%, RI 969 (calculated), RI 968 (verified)

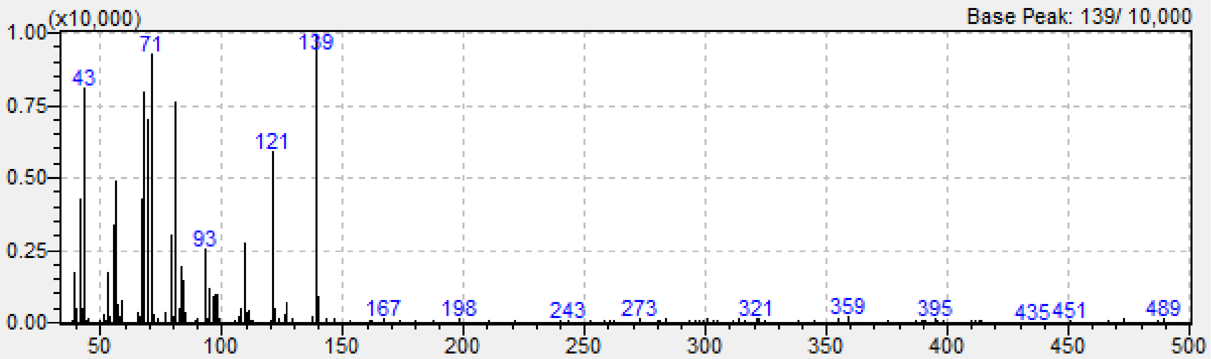

Heptanol, similarity MS 86%, RI 974 (calculated), RI 970 (verified)

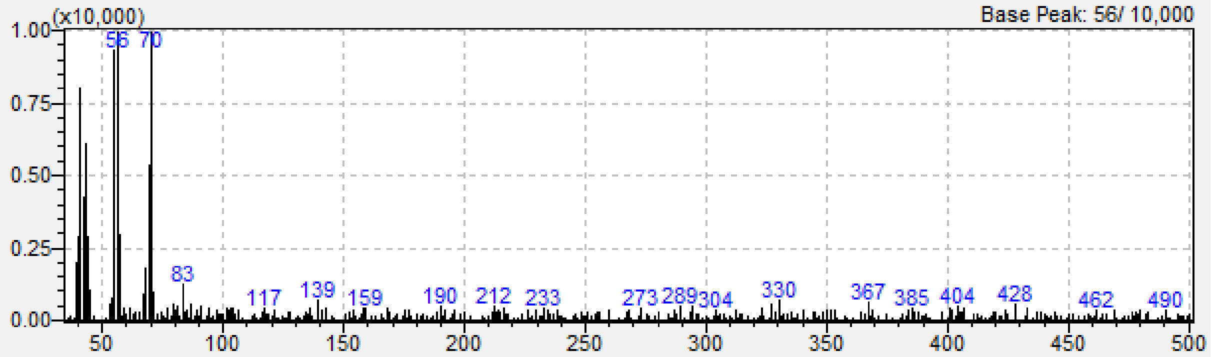

Hexyl acetate, similarity MS 89%, RI 1013 (calculated), RI 1012 (verified)

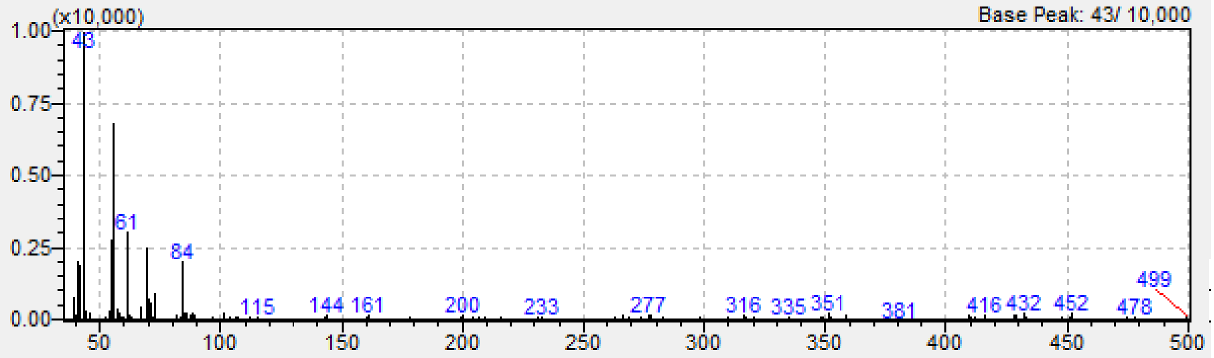

Unknown, RI 1018 (calculated)

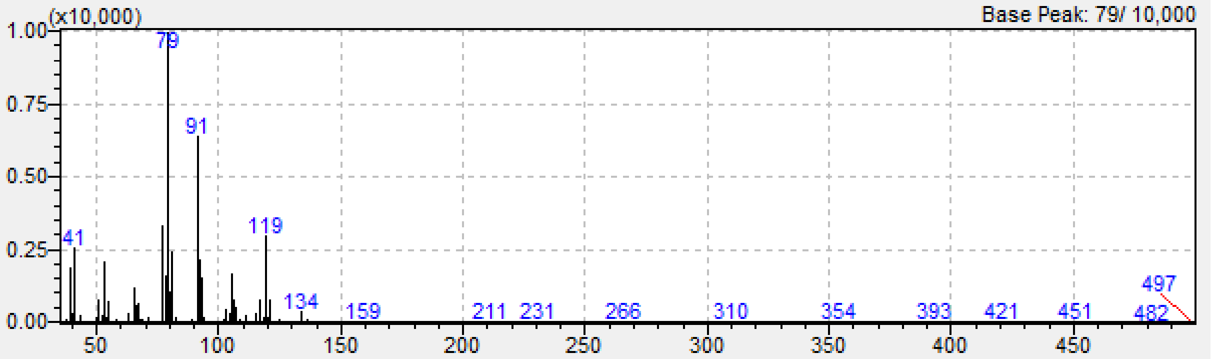

Limonene, similarity MS 93%, RI 1032 (calculated), RI 1030 (verified)

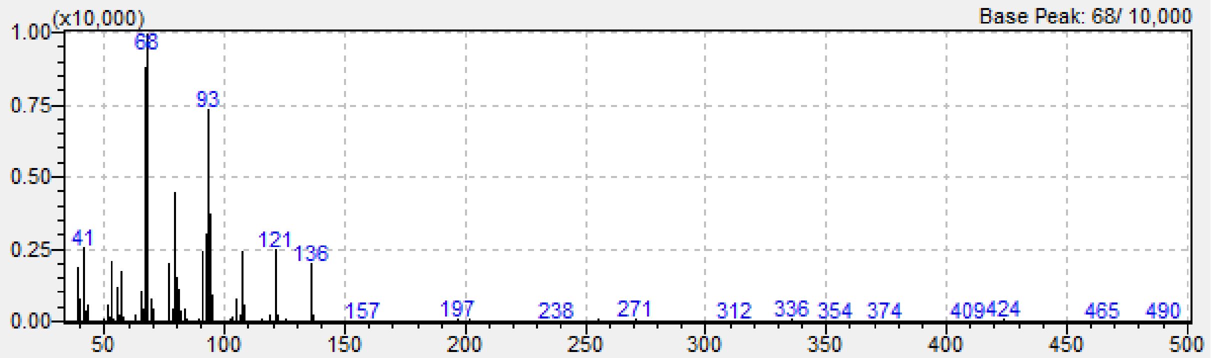

(Z)-β-Ocimene, similarity MS 93%, RI 1037 (calculated), RI 1035 (verified)

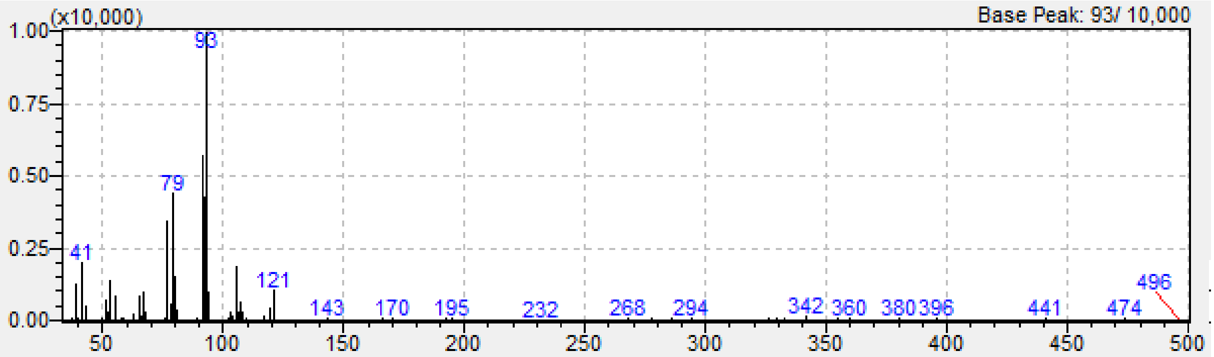

γ-Terpinene, similarity MS 89%, RI 1061 (calculated), RI 1058 (verified)

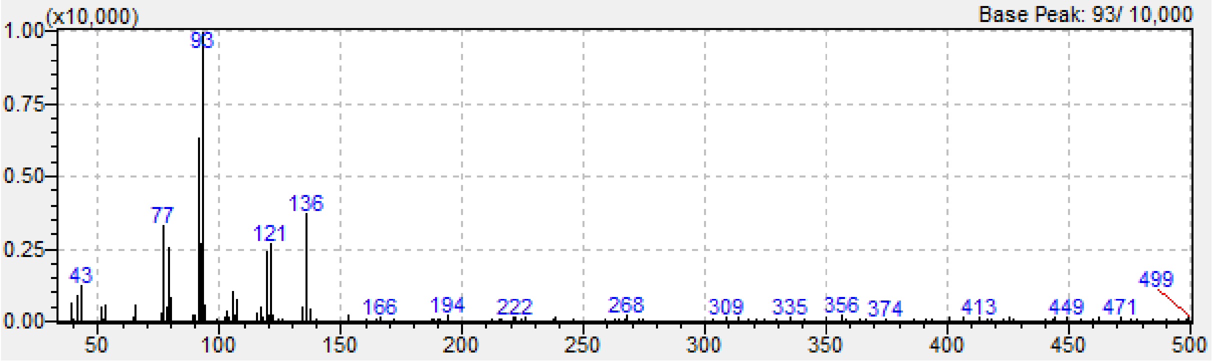

Isoamyl lactate, similarity MS 96%, RI 1069 (calculated), RI 1067 (verified)

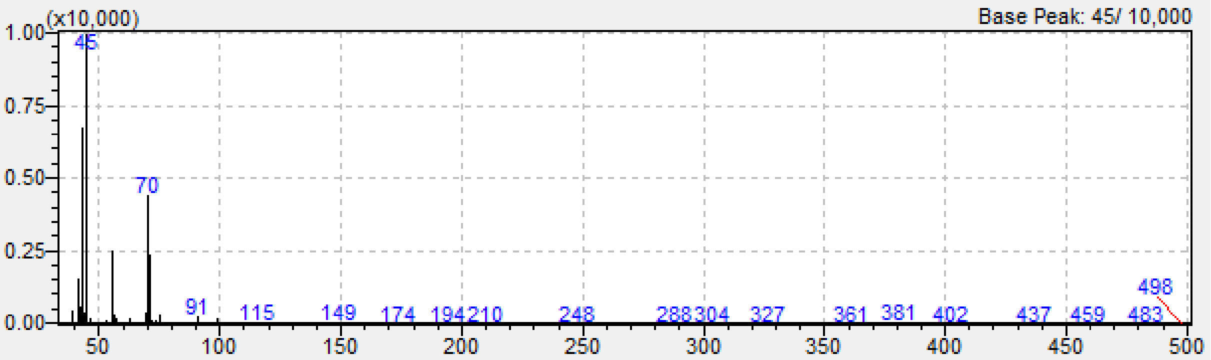

(Z)-Linalool furanoxide, similarity MS 91%, RI 1071 (calculated), RI 1069 (verified)

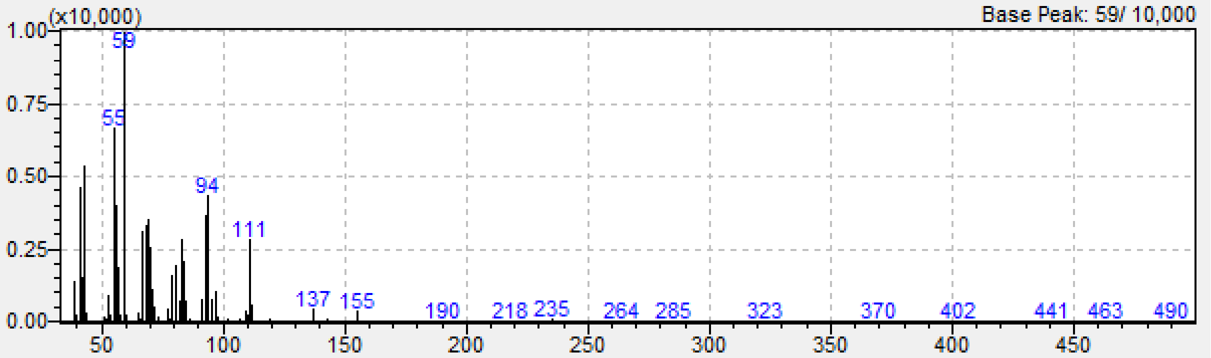

Caprylic acid, similarity MS 85%, RI 1183 (calculated), RI 1180 (verified)

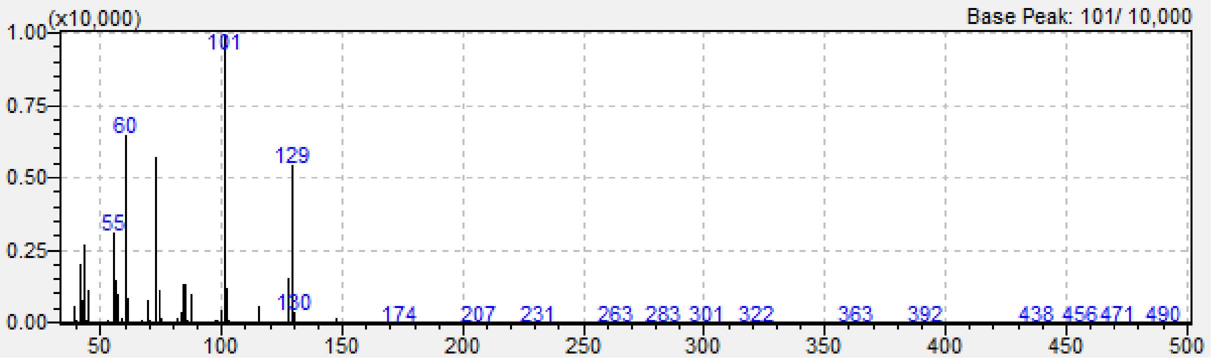

2-phenylethyl acetate, similarity MS 95%, RI 1257 (calculated), RI 1257 (verified)

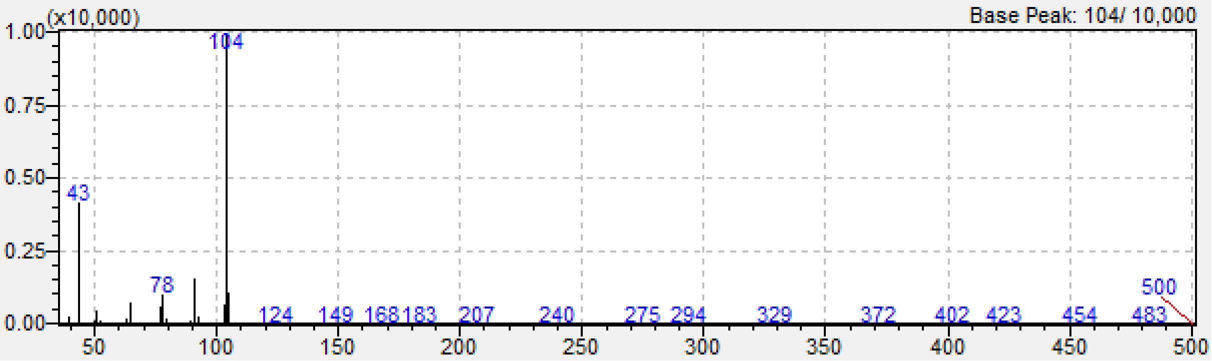

p-Ethylguaiacol, similarity MS 96%, RI 1278 (calculated), RI 1275 (verified)

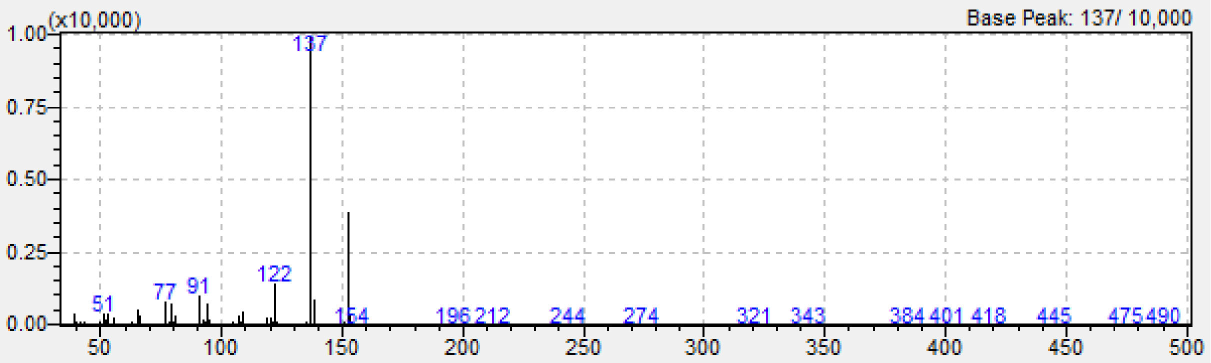

Unknown, RI 1299 (calculated)

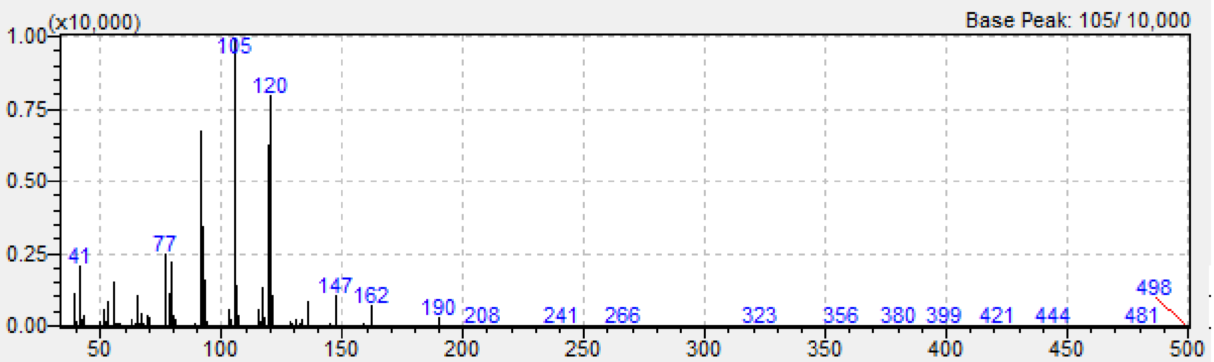

p-Vinylguaiacol, similarity MS 89%, RI 1316 (calculated), RI 1312 (verified)

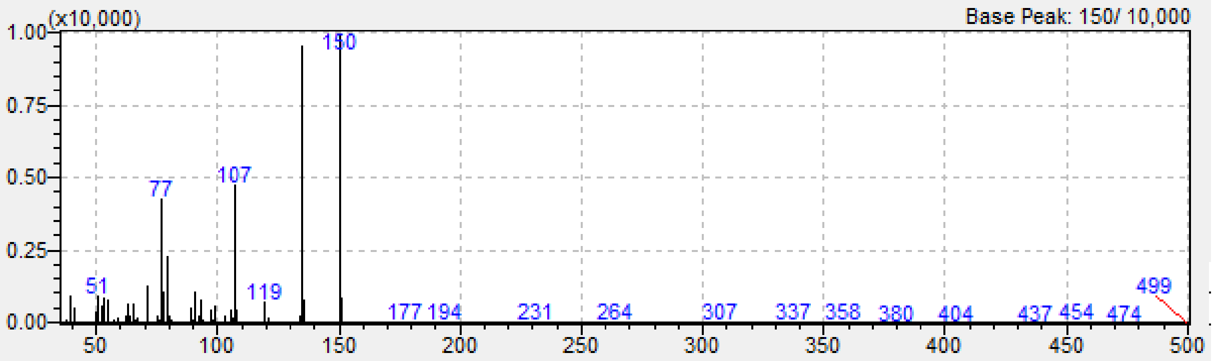

Unknown, RI 1454 (calculated)

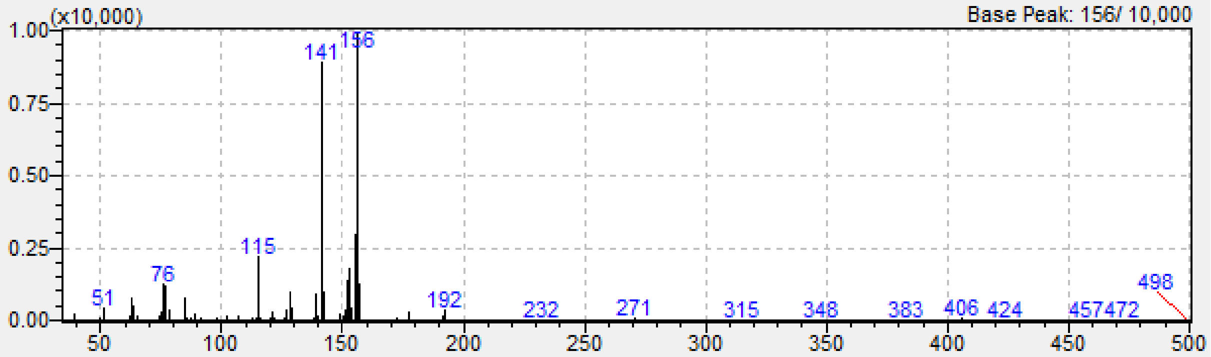

Unknown, RI 1483 (calculated)

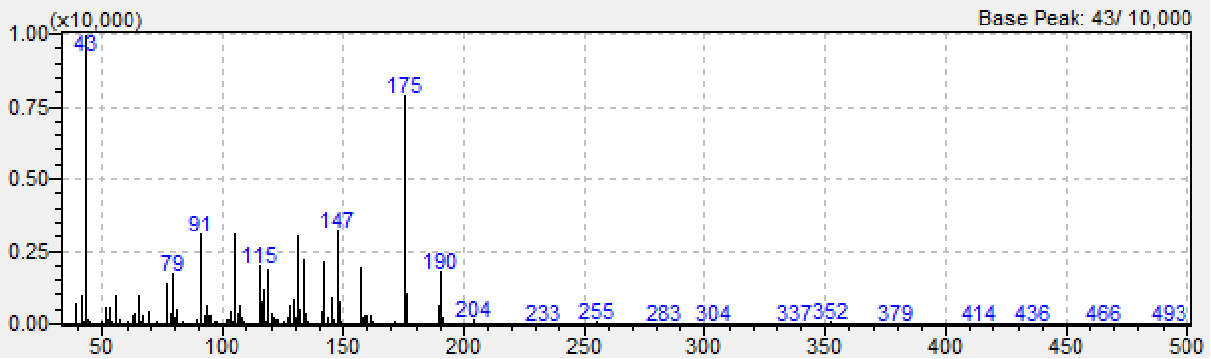

Myristaldehyde, similarity MS 96%, RI 1615 (calculated), RI 1614 (verified)

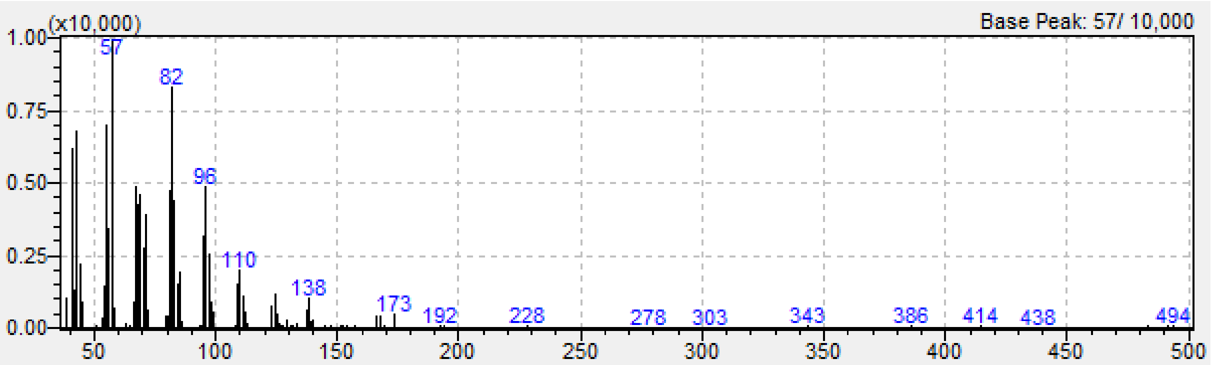

Supplementary Figures 2 Illustrating chromatograms of aromaprofile of measured wine by GC/FID (3 wine types – 2 Conventional wine, 1 Organic wine)

Sample 11 Conventional wine

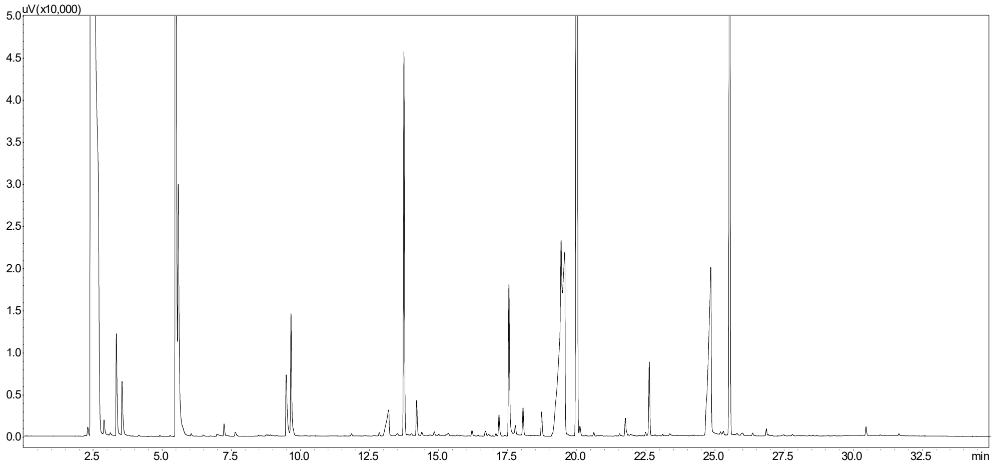

Sample 9 Conventional wine

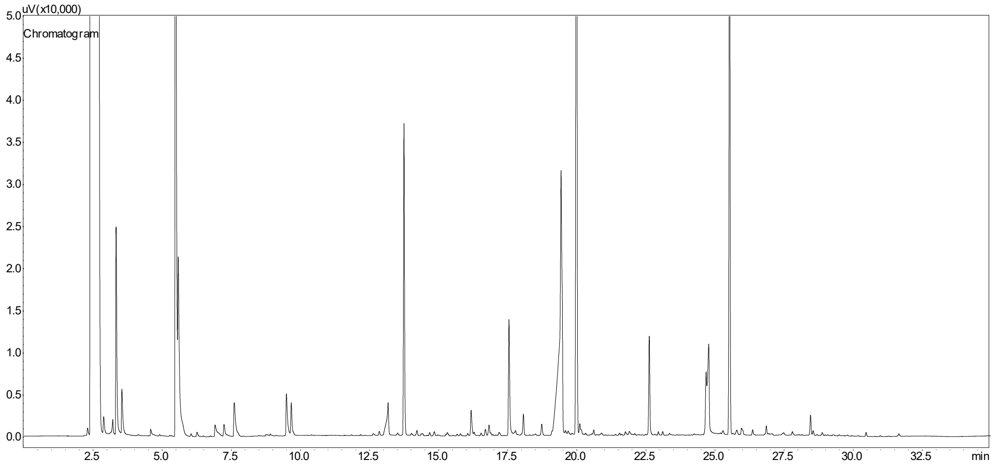

Sample 20 Organic wine

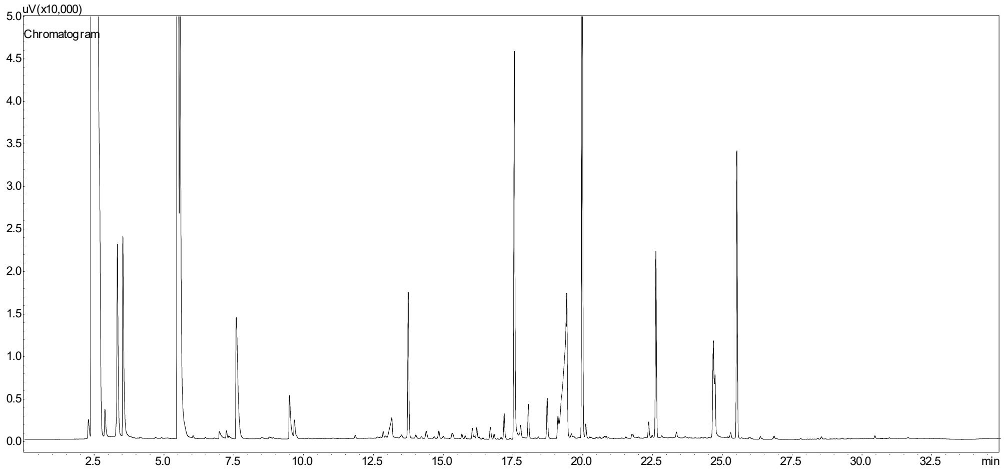

Supplementary Table 1 Average peak areas of the selected predictors (according to Table 2), expressed as % rel., and other compounds discussed in text of manuscript. The procedure for calculating % rel. is given in the text of manuscript (section Identification of relevant predictors)

		Conventional wine	Organic wine
**PREDICTORS**			
RI0813	Ethyl lactate	1,48	5,17
RI0877	Isoamyl acetate	0,80	0,51
RI0879	2-methylbutyl acetate	<0,01	0,11
RI0969	Linaloyl oxide	<0,01	0,01
RI0974	Heptanol	<0,01	0,01
RI1013	Hexyl acetate	0,21	0,03
RI1018	---	0,09	0,03
RI1032	Limonene	0,10	0,04
RI1037	(Z)-β-Ocimene	0,05	<0,01
RI1061	γ-Terpinene	0,03	0,01
RI1069	Isoamyl lactate	0,05	0,24
RI1071	(Z)-Linalool furanoxide	<0,01	0,04
RI1183	Caprylic acid	0,87	0,04
RI1257	2-phenylethyl acetate	0,12	0,05
RI1278	p-Ethylguaiacol	0,01	0,07
RI1299	---	0,04	<0,01
RI1316	p-Vinylguaiacol	0,01	0,01
RI1454	---	0,04	0,01
RI1483	---	0,06	0,04
RI1615	Myristaldehyde	0,02	<0,01
**OTHERS**			
	Hexanol	0,95	1,26
	Ethyl caprylate	23,92	12,56
